# Longan Polysaccharides with Covalent Selenylation Combat the Fumonisin B1-Induced Cell Toxicity and Barrier Disruption in Intestinal Epithelial (IEC-6) Cells

**DOI:** 10.3390/nu15214679

**Published:** 2023-11-04

**Authors:** Ya-Hui Yu, Xin-Huai Zhao

**Affiliations:** 1 School of Biology and Food Engineering, Guangdong University of Petrochemical Technology, Maoming 525000, China; 2 Research Centre of Food Nutrition and Human Healthcare, Guangdong University of Petrochemical Technology, Maoming 525000, China; 3 Maoming Branch, Guangdong Laboratory for Lingnan Modern Agriculture, Guangdong University of Petrochemical Technology, Maoming 525000, China; 4 College of Food Science, Northeast Agricultural University, Harbin 150030, China

**Keywords:** longan polysaccharides, selenylation, fumonisin B1, IEC-6 cells, cytotoxicity, barrier function, oxidative stress, apoptosis

## Abstract

In this study, the soluble, but non-digestible, longan (*Dimocarpus longan Lour.*) polysaccharides (LP) were extracted from dried longan fruits and then chemically selenylated to produce two selenylated products, namely SeLP1 and SeLP2, with different selenylation extents. The aim was to investigate their protective effects on rat intestinal epithelial (IEC-6) cells exposed to the food toxin fumonisin B1 (FB1). LP only contained total Se content of less than 0.01 g/kg, while SeLP1 and SeLP2 were measured with respective total Se content of up to 1.46 and 4.79 g/kg. The cell viability results showed that these two selenylated products were more efficient than LP in the IEC-6 cells in alleviating FB1-induced cell toxicity, suppressing lactate dehydrogenase (LDH) release, and decreasing the generation of intracellular reactive oxygen species (ROS). These two selenylated products were also more effective than LP in combating FB1-induced barrier disruption via increasing the transepithelial electric resistance (TEER), reducing the paracellular permeability, decreasing the mitochondrial membrane potential (MMP) loss, and maintaining cell barrier integrity by upregulating the tight-junction-related genes and proteins. FB1 caused cell oxidative stress and barrier dysfunction by activating the MAPK and mitochondrial apoptosis signaling pathways, while SeLP1 and SeLP2 could regulate the tMAPK- and apoptosis-related proteins to suppress the FB1-mediated activation of the two pathways. Overall, SeLP2 was observed to be more active than SeLP1 in the IEC-6 cells. In conclusion, the chemical selenylation of LP caused an activity enhancement to ameliorate the FB1-induced cell cytotoxicity and intestinal barrier disruption. Meanwhile, the increased selenylation of LP would endow the selenylated product SeLP2 with more activity.

## 1. Introduction

Fumonisins are well-known toxic metabolites produced by several Fusarium molds. It is agreed by the scientific community that fumonisins pose a serious threat to people’s health [1]. Fumonisin B1 (FB1), which is mainly detected in corn, is the most common and toxic member of the fumonisin family, characterized by hepatotoxicity, immunotoxicity, nephrotoxicity, and neurotoxicity, and it is also enterotoxic to animals [2,3,4]. The intestinal epithelial barrier is regarded as the first physical barrier and has a critical function in the body to protect against the potential invasion of various pathogens and toxins in circulation [5]. Recent studies have indicated that the ingestion of FB1 could inhibit the growth of intestinal epithelial cells, disrupt the expression of tight junction (TJ) proteins, regulate the immune responses of the intestine, and then cause inflammation and oxidative stress to induce barrier dysfunction in the intestinal epithelial cells [6,7,8,9]. It is thus critical to identify strategies to protect the intestine from FB1-induced barrier disruption. Special attention has been paid to food ingredients like polysaccharides, with potential activity to maintain intestinal barrier integrity, especially plant polysaccharides, due to their negligible safety risks and widespread existence in our daily diets. For example, the polysaccharides from *Portulaca oleracea* L. could maintain barrier function in the porcine intestinal epithelial cell monolayer via activating the EGF/EGFR signaling pathway [5], while those from *Gracilaria lemaneiformis* might enhance intestinal barrier function and maintain intestinal health [10]. Additionally, the polysaccharides from *Tremella fuciformis* and *Dendrobium huoshanense* can restore intestinal and mucosal barrier function [11,12]. However, whether polysaccharide modifications via chemical approaches might enhance the intestinal barrier function is rarely investigated; for instance, covalent polysaccharide selenylation incorporates the trace element Se into saccharide molecules.

Longan (*Dimocarpus longan Lour.*) is an edible fruit that is popular worldwide and has various health benefits [13]. Longan polysaccharides (LP) are some of the main bioactive substances with health benefits, including immuno-modulatory, anti-tumoral, and antioxidant effects and barrier protection in the intestine [13,14,15]. The activity of polysaccharides is considered to be related to their structural features, including the monosaccharide types, molecular weights, molecular structures, and chain glycosidic linkages, while the chemical modification of polysaccharides might change both the polysaccharide structures and activity [16]. Se is essential in the body, especially for the organic Se-containing substances like seleno-enzymes, namely thioredoxin reductase and glutathione peroxidase, critical to preventing cells and tissues from free-radical-induced injuries [17]. It is also well known that organic Se-containing substances have better bioavailability and absorption capacities, but are less toxic than inorganic Se [18]. The modification of polysaccharides via selenylation covalently introduces Se-containing chemical groups into polysaccharide molecules; thus, it is an efficient method to improve polysaccharide activity. Previous studies showed that selenylated polysaccharides were more active in enhancing the intestinal barrier function than polysaccharides themselves or inorganic Se [19,20], and Se-containing tea polysaccharides were more active in ameliorating ulcerative colitis in model mice via promoting intestinal barrier integrity and regulating important gut microbiota [21]. The chemical selenylation of LP also brought about a greater anti-cancer effect in two cell models (HT-29 and HCT-116 cells) [22,23]. Whether this LP selenylation causes activity changes in LP in the intestine to ameliorate the FB1-induced cell cytotoxicity and barrier damage on intestinal epithelial cells has not been examined yet. More importantly, how selenylated LP can mediate the cell barrier function remains to be clarified.

In the present study, two selenylated LP products with two selenylation extents, namely SeLP1 and SeLP2 having corresponding total Se content of 1.46 and 4.79 g/kg, were generated by reacting LP with the HNO_3_-Na_2_SeO_3_ system. Rat intestinal epithelial (IEC-6) cells were employed as a cell model, while the activity of SeLP1 and SeLP2 in the cells used to combat the FB1-induced cell toxicity and barrier disruption was identified, using unreacted LP as a control. This study aimed to identify whether the selenylated products acquired a better bioactivity capacity in the intestine to protect intestinal epithelial cells against FB1-caused toxicity and barrier destruction, and whether the selenylation extent of LP might impact the measured activity. The results could deepen our present knowledge about FB1 cell toxicity towards intestinal epithelial cells and identify the positive or negative effects of the conjugated trace element Se in polysaccharide molecules on polysaccharide activity in the intestine. In addition, the results could reveal whether the selenylated LP might be a potential functional food ingredient to maintain intestinal health.

## 2. Materials and Methods

### 2.1. Regents and Materials

Dried longan fruits were purchased from the market of Maoming City (Guangdong Province, China), while all used chemicals were of analytical grade. FB1 (≥98% purity by HPLC analysis) and pectinase (30 kU/g activity) were provided by the Aladdin Biochemical Technology Company Limited (Shanghai, China), while methyl thiazolyl tetrazolium (MTT) was produced by the Sigma-Aldrich Company Limited (Saint Louis, MO, USA). Lactate dehydrogenase (LDH), reactive oxygen species (ROS), mitochondrial membrane potential (MMP), and bicinchoninic acid (BCA) protein assay kits were all acquired from Beyotime Institute of Biotechnology (Shanghai, China), while trypsin–EDTA and cellulase (50 kU/g activity) were acquired from the Solarbio Science and Technology Company Limited (Beijing, China). The water used in the experiment was produced by a Milli-Q Plus system (Millipore Corp., New York, NY, USA).

The primary antibodies zonula occludens-1 (ZO-1) (AF5145), occludin (DF7504), claudin-1 (DF6919), JNK1/2/3 (AF6318), and phospho-JNK1/2/3 (AF3318) were obtained from Affinity Biosciences (Cincinnati, OH, USA). Cytochrome c (11940S), Bcl-2 (15071S), p38 MAPK (8690T), phospho-p38 MAPK (4511T), Bax (5023S), and caspases-3/9 (9662S/9508S) were the products of the Cell Signaling Technology (Shanghai) Biological Reagents Company Limited (Shanghai, China). Other primary antibodies, GAPDH (bs-10900R) and β-actin (bs-0061R), were acquired from the Bioss Biotechnology Company Limited (Beijing, China). In addition, horseradish-peroxidase-labeled goat anti-mouse IgG (ZB-2305) and goat anti-rabbit IgG (ZB-2301) were used as the secondary antibodies, provided by Zhongshan Golden Bridge Biotechnology (Beijing, China).

### 2.2. Extraction and Chemical Selenylation of LP

A reported procedure was used to extract LP [23]. In brief, the dried longan fruits were soaked in water and ground. The solid–liquid ratio and pH were 1:15 (*w*/*v*) and 4.5, respectively. Meanwhile, two enzymes, pectinase and cellulase, were added at 150 and 300 units/g of dried fruit, respectively, and the whole mixture was thereby kept at 50 °C for 4 h. Afterwards, the whole mixture was centrifuged at 8000× *g* for 10 min. The obtained supernatant was concentrated to 1/20 of the original volume and precipitated at 4 °C for 24 h using anhydrous ethanol with three volumes. The sediments (i.e., LP) were separated and washed with anhydrous ethanol in triplicate, followed by freeze-drying.

Based on a previous method, the HNO_3_-Na_2_SeO_3_ system was used to generate selenylated LP [23]. Briefly, LP of 500 mg was dissolved in 10 mL of 0.5% HNO_3_, and 25 or 75 mg of Na_2_SeO_3_ was added to the LP solution, while the whole solution was kept at 75 °C for 8 h. After centrifuging at 8000× *g* for 10 min, the whole solution was precipitated at 4 °C for 24 h using anhydrous ethanol with three volumes. The obtained sediments (i.e., SeLP1 and SeLP2) were rewashed with anhydrous ethanol and freeze-dried. Then, the Se content of LP, SeLP1, and SeLP2 was determined using a previous method [23].

### 2.3. Cell Culture and Assay of Cell Viability

Compared with Caco-2 cells, untransformed rat small intestinal epithelial (IEC-6) cells have a transepithelial electric resistance value of about 50 Ω cm^2^, which is very close to that of the human small intestine (40 Ω cm^2^), while they have additional similar physiological characteristics [24,25]. Therefore, IEC-6 cells were chosen in this study to investigate intestinal epithelial barrier function. The IEC-6 cells were provided by the American Type Culture Collection (Rockville, MD, USA). In this study, the cells were cultured at 37 °C in a humidified 5% CO_2_ incubator using complete Dulbecco’s modified Eagle’s medium (DMEM) (Sigma-Aldrich, Saint Louis, MI, USA). Additionally, 10% fetal bovine serum from Wisent Incorporated (Montreal, QC, Canada), 1.5 g/L NaHCO_3_, 1% sodium pyruvate, and 100 U/mL penicillin/streptomycin were used in the medium preparation.

The MTT assay was used to evaluate the effect of FB1 on IEC-6 cells and the effects of LP, SeLP1, and SeLP2 on the FB1-induced cells. Specifically, cells with a density of 5 × 10^3^ cells/well were inoculated into 96-well plates for 24 h and incubated for 12 h in serum-free media. After discarding the medium, the cells were treated with FB1 (10–160 μmol/L) for 24 and 48 h. Otherwise, the cultured cells were exposed to the medium or polysaccharide samples (LP, SeLP1, and SeLP2) at 2.5–40 μg/mL for 24 and 48 h, followed by FB1 treatment at 40 μmol/L for 48 h. Discarding the medium, 100 μL MTT solution (the complete medium of 90 μL plus 5 mg/mL MTT of 10 μL) was added to each well, while the cells were incubated for 4 h. Dimethyl sulfoxide (DMSO) of 150 μL was added to each well after discarding the medium. A microplate reader (Bio-Rad Laboratories, Hercules, CA, USA) was thus employed to measure the optical density at 490 nm. Cell viability was calculated as a percentage of the control cells [23], while the cell viability of the control cells without FB1 or polysaccharide sample treatment was set at 100%.

### 2.4. Assay of Transepithelial Electrical Resistance

The 12-well transwell membrane inserts (Corning, Kennebunk, ME, USA) had well and pore diameters of 12 mm and 0.4 μm, respectively. The cells were seeded at a cell density of 1 × 10^5^ cells/well, cultured for 21 d with medium, and replaced every 2 d. A Millicell-ERS2 Volt-Ohm Meter (Millipore, Bedford, MA, USA) was used to measure the transepithelial electric resistance (TEER) value of the confluent cell monolayer until a TEER value of 50 Ω cm^2^ was reached. Afterward, the cells were incubated for 12 h in serum-free medium, treated with the medium or polysaccharide samples at 5–10 μg/mL for 24 and 48 h, and injured by FB1 (40 μmol/L) for 48 h. The TEER value was calculated as previously described [26] and reported as the percentage value of the control cells.

### 2.5. Assay of Paracellular Permeability

The effect of polysaccharide samples on the paracellular permeability of the cell monolayer was evaluated as follows. The cells (1 × 10^5^ cells/well) were inoculated into the 12-well transwell membrane inserts to a TEER value of 50 Ω cm^2^. Afterward, the cells were incubated for 12 h in a serum-free medium, treated with the medium or polysaccharide samples at 5–10 μg/mL for 24 and 48 h, and then injured by FB1 (40 μmol/L) for 48 h. Then, 0.5 mg/mL 4 kDa fluorescein isothiocyanate (FITC)–dextran (FD-4) (Sigma-Aldrich, Saint Louis, MI, USA) of 0.5 mL was added to each apical compartment and incubated for 4 h. The medium from the basal compartment was added to 96-well plates, whilst a microplate reader was used to detect the resultant fluorescent intensity using excitation/emission wavelengths of 490/520 nm. The fluorescence level (recorded in relative fluorescence units) was calculated and expressed as previously described [26]. The control cells were set with an FD-4 cumulative transport value of 100%.

### 2.6. Measurements of LDH Release, Intracellular ROS, and MMP

The cells (5 × 10^3^ cells/well) were inoculated into 96-well plates for 24 h, incubated in serum-free medium for 12 h, treated by the medium or polysaccharide samples at 5–10 μg/mL for 24 and 48 h, and injured by FB1 (40 μmol/L) for 48 h. Using the kit manual, cell supernatants of 100 μL were collected, added with 60 μL LDH detection working solution, and cultured for 30 min at 25 °C. The microplate reader was used to detect the optical density at 490 nm, which was reported as a percentage of the control cells. The control cells were set with an LDH release value of 100%.

The cells (1 × 10^5^ cells/well) were inoculated into 6-well plates for 24 h, incubated for 12 h in serum-free medium, treated by the medium or polysaccharide samples at 5–10 μg/mL for 24 and 48 h, and then injured by FB1 (40 μmol/L) for 48 h. The cells were collected and washed with phosphate-buffered saline (PBS) (Solarbio, Beijing, China) (pH 7.2, 10 mmol/L) twice, while 1 mL ROS fluorescent probe DCFH-DA (2′,7′-dichlorodihydrofluorescein diacetate, 5 μmol/L) was added and cultured at 37 °C for 20 min. The cells were inoculated into 96-well plates. The microplate reader was used to detect the fluorescence intensity at excitation/emission wavelengths of 488/525 nm. The result was calculated relative to the control cells’ intracellular ROS level (set with an intracellular ROS level of 100%).

To assay MMP loss, the cells treated as above were collected and washed, while 1 mL MMP fluorescent probe JC-1 was added and cultured at 37 °C for 20 min. The cells were inoculated into 96-well plates. In this assay, the microplate reader was applied to detect the resultant fluorescence intensity using excitation and emission wavelengths of 488/525 and 525/590 nm, respectively. The red/green fluorescence ratio (fluorescence level) was calculated and expressed as previously described [23].

### 2.7. Reverse Transcription Quantitative Real-Time PCR Assay

The cells (1 × 10^5^ cells/well) were inoculated into 6-well plates for 24 h and then incubated for 12 h in serum-free medium, treated by the medium or polysaccharide samples (10 μg/mL) for 24 h, and injured by FB1 (40 μmol/L) for 48 h. The RNAprep pure cell kit from the Tiangen Biotech Company Limited (Beijing, China) was used to extract total RNA, while the complementary DNA (cDNA) was used in the reverse transcription of the RNA using the NovoScript^®^ two-step RT-PCR kit from the Novoprotein Biotech Company Limited (Suzhou, China). Afterward, the cDNA was amplified using the NovoScript^®^ SYBR qPCR SuperMix Plus (Novoprotein, Suzhou, China) and Biosystems StepOnePlus real-time PCR system from the Life Technologies Corporation (Carlsbad, CA, USA). The results were calculated using the 2^−ΔΔCt^ method, as previously described [27]. The sequences of the used primers are listed in Table 1, while the internal standard was the GAPDH housekeeping gene.

### 2.8. Western Blotting Assay

The cells at 5 × 10^5^ cells/flask were seeded onto a 25 cm^2^ cell culture flask for 24 h, incubated for 12 h in serum-free medium, treated by the medium or polysaccharide samples (10 μg/mL) for 24 h, and injured by FB1 (40 μmol/L) for 48 h. The 300 μL radio-immunoprecipitation assay (RIPA) lysis buffer (containing protease and phosphatase inhibitor cocktail) (Beyotime, Shanghai, China) was added and lysed for 20 min at 4 °C. The lysed solution was centrifuged at 12,000× *g* for 5 min, while the obtained supernatants were evaluated for their protein concentrations using the BCA protein analysis kit. An equal amount of the protein sample was thereby separated using an SDS-PAGE gel, electro-transferred to the nitrocellulose membranes, and incubated successively with 5% skimmed milk (2 h, 37 °C), primary antibodies (1:1000 dilution, 12 h, 4 °C), and secondary antibodies (1:5000 dilution, 1 h, 37 °C). Protein bands were finally assayed using Image Quant LAS 500 (Fujifilm, Tokyo, Japan). The Image J 2x software of the National Institutes of Health (Bethesda, MD, USA) was applied to quantify the targeted bands. The internal standard (β-actin or GAPDH) was also used to normalize the band density.

### 2.9. Statistical Analyses

The reported data were expressed as the means or means ± standard deviations, while the Social Sciences 16.0 software package from SPSS Incorporated (Chicago, IL, USA) and Duncan’s multiple range tests were used to analyze significant differences (*p* < 0.05) between the mean values of the individual group.

## 3. Results

### 3.1. Effects of Polysaccharide Samples on FB1-Induced Cytotoxicity

**Figure 1:**
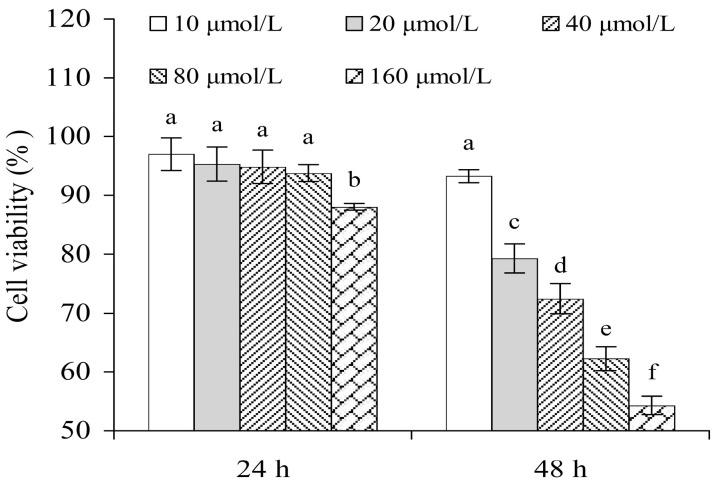
Effect of FB1 (10–160 μmol/L) with 24–48 h treatment time on viability of IEC-6 cells. Significant variances in the mean values are denoted above the columns by various lowercase letters (*p* < 0.05).

FB1 was assessed for its toxic effect on IEC-6 cells at the targeted five separate concentrations (10–160 μmol/L) (Figure 1), as the FB1 concentration at the intestinal brush border in humans was expected to be 1–138 μmol/L [28]. Obviously, FB1 dose- and time-dependently exerted a toxic effect on the cells, leading to viability values of 88.0–97.0% (24 h) or 54.3–93.3% (48 h). When FB1 of 40 μmol/L was used to injure the cells for 48 h, it caused a viability value near 72%. An FB1 dose of 40 μmol/L and treatment time of 48 h were selected to induce cell injury in the subsequent assays in this study.

**Figure 2:**
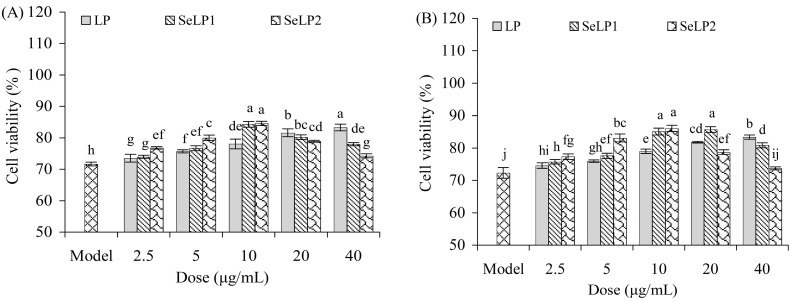
Effect of polysaccharide products (LP, SeLP1, and SeLP2, 2.5–40 μg/mL) on viability of IEC-6 cells with treatment times of 24 (**A**) and 48 h (**B**), followed by 40 μmol/L FB1 exposure for 48 h. The model cells were only treated with FB1 of 40 μmol/L for 48 h. Significant variances in the mean values are denoted above the columns by various lowercase letters (*p* < 0.05).

The analysis data revealed that the natural LP contained Se less than 0.01 g/kg, while the two synthetically selenylated products, SeLP1 and SeLP2, had corresponding Se content of 1.46 and 4.79 g/kg. Meanwhile, pretreatment of the cells with these polysaccharide samples could ameliorate the FB1-induced viability reduction, because all pretreated cells showed viability values higher than 72% (Figure 2A,B), suggesting that these samples had the ability to combat the FB1-induced cell toxicity. In detail, the cells pretreated with LP (2.5–40 μg/mL) for 24–48 h before the FB1 injury had viability values of 73.5–83.3% (24 h) or 74.5–84.4% (48 h), while those pretreated with SeLP1 for the same doses and time periods showed viability values of 74.0–84.3% (24 h) or 75.7–85.6% (48 h). More importantly, the cells pretreated with SeLP2 for the same doses and time periods had viability values of 74.3–84.6% (24 h) or 73.7–86.0% (48 h). Therefore, SeLP2 and LP showed the highest and lowest activity to increase cell viability, respectively, while polysaccharide samples at 10 μg/mL caused the highest cell viability. Polysaccharide dose levels of 5 and 10 μg/mL and treatment times of 24 and 48 h were thus used in the later experiments of this study.

**Figure 3:**
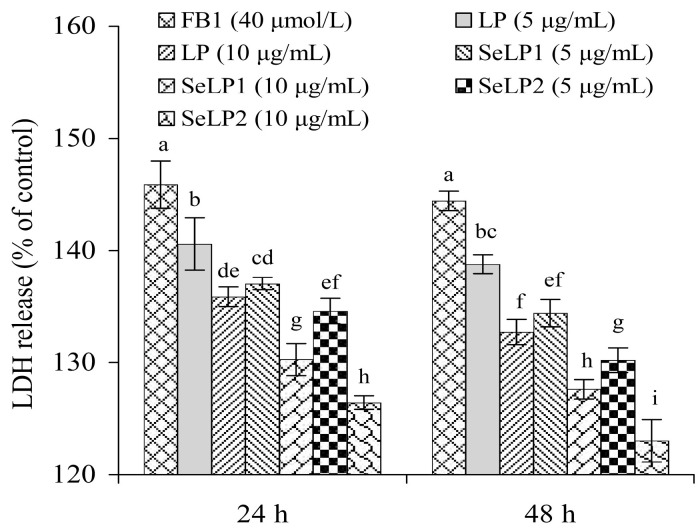
Lactate dehydrogenase (LDH) release of IEC-6 cells exposed to the medium or polysaccharide products (LP, SeLP1, and SeLP2, 5–10 μg/mL) for 24 and 48 h, followed by 40 μmol/L FB1 exposure for 48 h. Significant variances in the mean values are denoted above the columns by various lowercase letters (*p* < 0.05).

Cell injury accompanies the membrane disruption of cells, leading to more lactate dehydrogenase (LDH) release. Compared to the control cells, the FB1-alone-treated cells had a distinct increase in LDH release (145.9% versus 100.0%) (Figure 3). When the cells were pretreated by LP (5 and 10 μg/mL) before the FB1 exposure, the measured LDH release was decreased to 135.9–140.6% (24 h) or 132.7–138.8% (48 h). Additionally, the SeLP1-pretreated cells showed LDH release of 130.3–137.0% (24 h) or 127.6–134.4% (48 h), while the SeLP2-pretreated cells had significantly reduced LDH release of 126.4–134.6 (24 h) or 123.0–130.2% (48 h). These data suggest that the pretreatment of the cells by these polysaccharide samples before the FB1 injury could suppress LDH release. SeLP1 was more efficient than LP in suppressing LDH release in the cells, while SeLP2 showed higher activity than SeLP1. SeLP2 exhibited the highest activity to alleviate the FB1-induced cell viability decrease and LDH release increase (Figure 2 and Figure 3). Thus, both chemical selenylation and a higher selenylation extent provided SeLP2 with a greater activity increase.

### 3.2. Effect of Polysaccharide Samples on Cell Barrier Integrity

**Figure 4:**
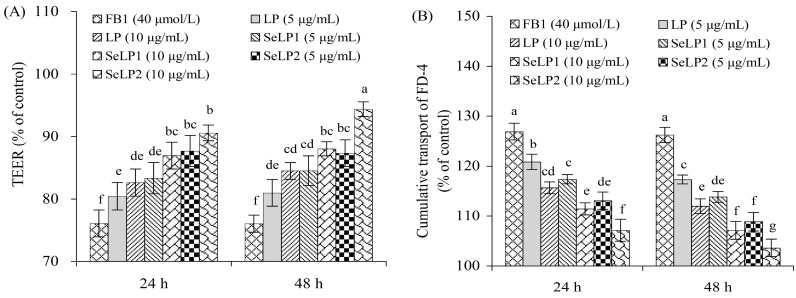
Transepithelial resistance (TEER) values (**A**) and paracellular permeability (**B**) of IEC-6 cells treated by the medium or polysaccharide products (LP, SeLP1, and SeLP2, 5–10 μg/mL) for 24 and 48 h, followed by 40 μmol/L FB1 exposure for 48 h. Significant variances in the mean values are denoted above the columns by various lowercase letters (*p* < 0.05).

FB1 was observed in this study to damage the barrier function of IEC-6 cells via decreasing the transepithelial electric resistance (TEER) value but increasing the FD-4 cumulative transport value (Figure 4). As shown in Figure 4A, the TEER value of the FB1-alone-treated cells decreased to 76.1%, suggesting injured barrier integrity. Meanwhile, LP, SeLP1, and SeLP2 (at 5 and 10 μg/mL) consistently led to higher TEER values (i.e., improved barrier integrity). The cells pretreated by LP showed TEER values of 80.4–82.6% (24 h) or 80.9–84.5% (48 h). Meanwhile, the cells pretreated with SeLP1 and SeLP2 showed further increases in TEER values (83.3–87.0% and 87.7–90.6%, 24 h; or 84.5–88.0% and 87.3–94.4%, 48 h). Furthermore, the FB1-alone-treated cells showed an increased fluorescein isothiocyanate (FITC)–dextran (FD-4) flux value (126.9%), suggesting enhanced paracellular permeability (Figure 4B). However, LP, SeLP1, and SeLP2 could reduce the FD-4 flux values and ameliorate the FB1-caused permeability increase. In detail, the cells pretreated by the LP had FD-4 flux values of 115.7–120.9% (24 h) or 112.0–117.3% (48 h), while the cells pretreated with SeLP1 and SeLP2 showed respective FD-4 flux values of 111.5–117.4.0% and 107.2–113.1% (24 h) or 107.1–113.8% and 103.6–108.9% (48 h). The cells pretreated with polysaccharide samples were thus suggested to show an improvement in barrier integrity, regarding the model cells (i.e., the FB1-alone-treated cells). The results also demonstrated that LP and SeLP2 had the weakest and strongest ability to enhance cell barrier integrity, disclosing again that the used chemical selenylation could endow LP with a greater ability in the cells to increase TEER, but it decreased paracellular permeability, while a higher selenylation extent also brought about an activity increase.

### 3.3. Effect of Polysaccharide Samples on FB1-Induced Intracellular ROS Generation and MMP Loss

**Figure 5:**
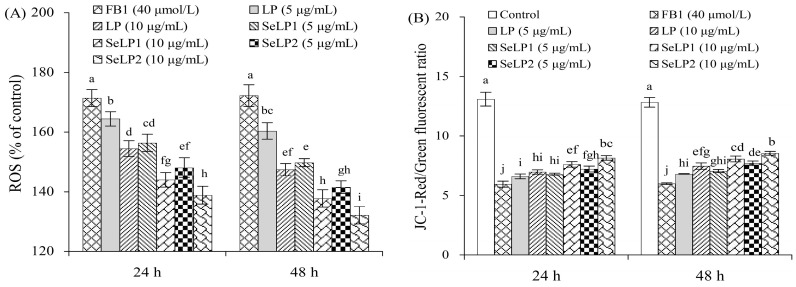
Reactive oxygen species (ROS) levels (**A**) and mitochondrial membrane potential (MMP) loss (**B**) of IEC-6 cells treated by the medium or polysaccharide products (LP, SeLP1, and SeLP2, 5–10 μg/mL) for 24 and 48 h, followed by 40 μmol/L FB1 exposure for 48 h. Significant variances in the mean values are denoted above the columns by various lowercase letters (*p* < 0.05).

FB1 exposure might cause cytotoxicity through oxidative stress. The reactive oxygen species (ROS) production in the cells was thus evaluated (Figure 5A). Compared to the control cells (ROS level of 100%), the ROS level of the FB1-alone-treated cells was distinctly increased to 172.2%, indicating that FB1 caused pro-oxidation and thus elevated ROS generation. LP (5 and 10 μg/mL) caused ROS levels of 154.5–164.4% (24 h) or 147.4–160.4% (48 h), while SeLP1 at the same doses led to lower ROS levels (144.0–156.4%, 24 h; or 137.7–149.7%, 48 h). Additionally, SeLP2 at the same doses decreased ROS production (138.8–148.1%, 24 h; or 132.1–141.5%, 48 h). The polysaccharide samples thus eliminated oxidative stress partly and then reduced ROS production. SeLP1, especially SeLP2, had a stronger ability than LP to eliminate cellular oxidative stress.

FB1 exposure also caused cell mitochondrial membrane potential (MMP) loss (Figure 5B). The control cells at 24 and 48 h had red/green fluorescence rations of about 13.1 and 12.8, respectively. The FB1-alone-treated cells showed a reduced ratio of red/green fluorescence (about 5.9), indicating the occurrence of MMP loss. When the cells were pretreated with LP (5–10 μg/mL) before the FB1 exposure, the measured ratios were increased to 6.6–7.0 (24 h) or 7.1–7.5 (48 h), demonstrating less MMP loss. The SeLP1-pretreated cells had ratio values of 6.8–7.6 (24 h) or 7.1–8.1 (48 h), while the SeLP2-pretreated cells showed ratio values of 7.2–8.2 (24 h) or 7.7–8.6 (48 h), illustrating that the FB1-casued MMP loss was significantly reduced. All the results consistently proved that the polysaccharide samples could ameliorate the FB1-caused damage on mitochondrial membranes, and the conducted selenylation could endow LP with a greater capacity to protect the cells from the mentioned damage; moreover, the higher selenylation level of LP resulted in stronger activity towards the cells.

Generally, the enhanced but unusual generation of ROS in cells can damage mitochondrial membranes and trigger cell apoptosis. Based on these results (Figure 5), it was inferred that FB1 might induce barrier disruption by mediating the cells’ MAPK and mitochondria-mediated apoptosis pathways. It was thus necessary to clarify whether polysaccharide samples exerted barrier protection by regulating these signaling pathways.

### 3.4. Effect of Polysaccharide Samples on the Expression of the TJ-Related Genes and Proteins

**Figure 6:**
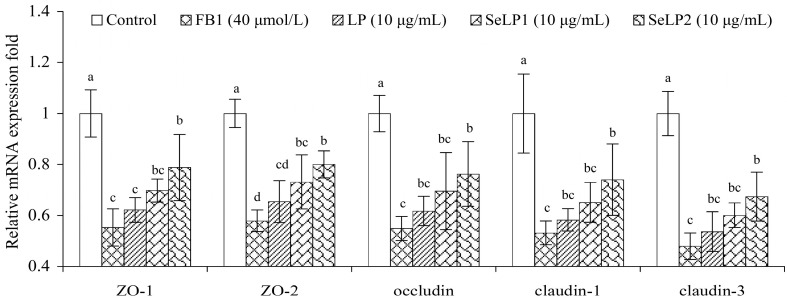
Changes in the expression of tight junction (TJ)-related genes in IEC-6 cells exposed to the medium or polysaccharide products (LP, SeLP1, and SeLP2, 10 μg/mL) for 24 h, followed by 40 μmol/L FB1 exposure for 48 h. Significant variances in the mean values are denoted above the columns by various lowercase letters (*p* < 0.05).

To further clarify the ability of these polysaccharide samples in the IEC-6 cells to alleviate the FB1-caused barrier dysfunction, five TJ-related genes (ZOs-1/2, occludin, and claudins-1/3) were analyzed for their expression changes (Figure 6). The relative expression levels of these five genes in the control cells were all defined as 1.0 fold. The FB1-alone-treated cells had lower relative expression levels for these TJ-related genes (0.5–0.6 folds), while the LP-pretreated cells at 10 μg/mL mostly showed upregulated gene expression (0.5–0.8 folds). Moreover, SeLP1 and especially SeLP2 induced much higher expression for these five TJ-related genes than LP, indicating once more that the used chemical selenylation and a higher selenylation extent enhanced the activity of LP.

**Figure 7:**
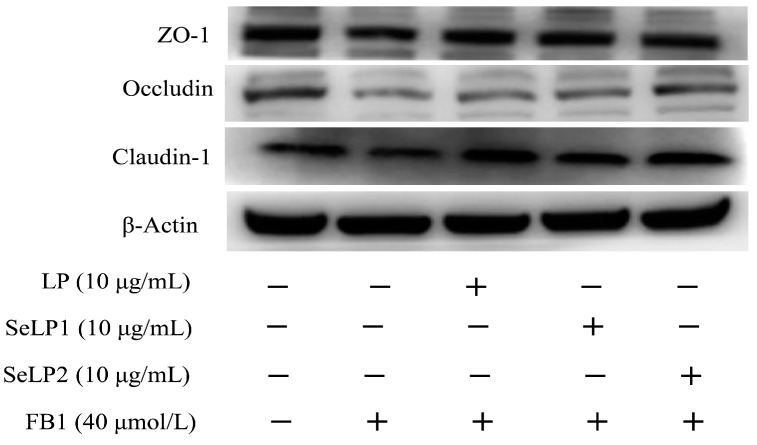
Expression of ZO-1, occludin, and claudin-1 in IEC-6 cells treated by the medium or polysaccharide products (LP, SeLP1, and SeLP2, 10 μg/mL) for 24 h, followed by 40 μmol/L FB1 exposure for 48 h.

The Western blotting results verified that the polysaccharide samples regulated the expression of the three TJ-related proteins (ZO-1, occludin, and claudin-1) in the cells (Figure 7 and Table 2). In brief, the FB1-alone-treated cells showed lower levels of relative expression for these three proteins (0.5–0.6 folds), while the cells pretreated with the polysaccharide samples (10 μg/mL) showed higher relative expression for these three proteins (0.6–0.9 folds). SeLP1 and especially SeLP2 were more effective than LP in upregulating the expression of ZO-1, occludin, and claudin-1. These results (Figure 6 and Figure 7) prove again that the employed chemical selenylation conferred LP with increased activity, and the higher selenylation extent also caused an activity increase. Subsequently, the selenylated products more efficiently resisted the FB1-induced barrier dysfunction.

### 3.5. Effect of Polysaccharide Samples on the Expression of MAPK- and Apoptosis-Related Proteins

**Figure 8:**
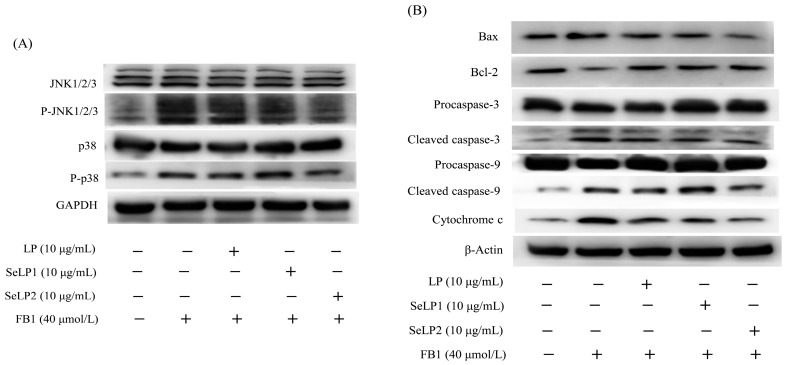
Expression of the MAPK-related (**A**) and apoptosis-related (**B**) proteins in IEC-6 cells treated by the medium or polysaccharide products (LP, SeLP1, and SeLP2, 10 μg/mL) for 24 h, followed by 40 μmol/L FB1 exposure for 48 h.

The results also indicated that the protein expression of four key proteins, namely p38/p-p38 and JNK/p-JNK (involved in MAPK signaling pathway), was altered in response to cell treatment with FB1 or the polysaccharide samples (Figure 8A and Table 3). Compared to the control cells, the FB1-alone-treated cells had upregulated expression for p-p38/p38 (2.5-folds) and p-JNK/JNK (2.0-folds), while the cells pretreated with the polysaccharide samples (10 μg/mL) showed less upregulated expression for p-p38/p38 (1.5–2.2 folds) and p-JNK/JNK (1.3–1.6 folds). In theory, upregulated p-p38/p38 and p-JNK/JNK expression can activate the MAPK signaling pathway. It was confirmed that FB1 induced barrier dysfunction via activating the MAPK signaling pathway, while the polysaccharide samples (especially SeLP2) ameliorated the FB1-induced barrier disruption via suppressing MAPK signaling pathway activation.

Furthermore, it was also found that the cells treated with the polysaccharide samples had regulated expression for seven apoptosis-related proteins, namely Bax, Bcl-2, caspase-3, caspase-9, cytochrome c, cleaved caspase-3, and cleaved caspase-9 (Figure 8B and Table 3). In total, the FB1-alone-treated cells had upregulated expression of the four pro-apoptotic proteins Bax, cleaved caspase-3, cleaved caspase-9, and cytochrome c (with respective 1.7-, 1.9-, 2.2-, and 1.9-fold increases), together with downregulated expression of anti-apoptotic protein Bcl-2 (0.6-fold decrease). FB1 was thus considered to induce cell apoptosis and cause barrier dysfunction via activating the mitochondrial apoptosis pathway. However, LP, SeLP1, and SeLP2 at 10 μg/mL resulted in less regulation for Bax, cleaved caspase-3, cleaved caspase-9, cytochrome c, and Bcl-2, which were detected with 1.1–1.5, 1.5–1.9, 1.5–1.9, 1.5–1.9, and 0.7–0.9 fold expression levels, respectively. The polysaccharide samples were capable of suppressing the FB1-induced apoptosis through inactivating the mitochondrial apoptosis pathway. In short, SeLP1 and especially SeLP2 were more effective than LP in regulating the expression levels of these MAPK- and apoptosis-related proteins, suggesting that the used chemical selenylation and a higher selenylation extent contributed to the increased activity of the modified LP.

**Figure 9:**
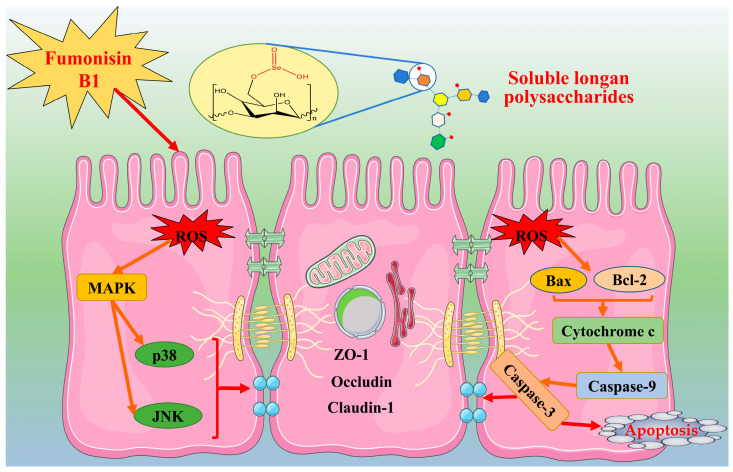
The underlying pathways by which the selenylated longan polysaccharides (LP) protect IEC-6 cells from FB1-induced barrier disruption.

Overall, it was thus verified that FB1 induced critical barrier dysfunction in IEC-6 cells by activating both the MAPK and mitochondrial apoptosis signaling pathways, while the assessed polysaccharide samples, particularly SeLP2, exerted barrier protection on the cells by suppressing the activation of these pathways, as described in Figure 9.

## 4. Discussion

FB1 exposure disrupts the redox balance of cells and tissues in the body; thus, critical mitochondrial respiration leads to the generation of excessive toxic aerobic free radicals that can induce oxidative stress and cell toxicity [2]. Previous results showed that FB1 exposure in human gastric epithelial (GES-1), kidney tubular epithelial (HK-2), and monkey kidney (MARC-145) cells caused cytotoxicity and cell death [29,30,31], while FB1 in human liver hepatoblastoma (HepG2) cells led to oxidative stress [32]. FB1 could also induce cytotoxicity proliferation inhibition and promote cell death in IPEC-J2 cells from the porcine intestine [33]. Consistent with these reported results, this study also revealed that FB1 could dose- and time-dependently exert cytotoxicity on IEC-6 cells, causing growth inhibition and MMP loss, promoting LDH release, and enhancing ROS production.

It was proven in previous studies that another toxin, lipopolysaccharide (LPS), could induce intestinal barrier injury in the Caco-2 monolayer or rat model by activating the MAPK signaling pathway [34,35], and indomethacin in tumor-bearing mice and IEC-6 cells could injure the intestinal barrier and decrease TJ proteins via activating the MAPK signaling pathway [36]. Moreover, it was verified that cyclophosphamide could damage the intestinal barrier and downregulate TJ proteins in mice through activating the p38/JNK signaling pathway [37], and LPS could cause barrier dysfunction in IPEC-J2 cells via promoting p38/JNK phosphorylation and cell apoptosis [38]. Cell apoptosis is thus regarded to be involved in the barrier dysfunction of cells. For example, previous results showed that the *Clostridium perfringens* beta2 toxin was able to disrupt the barrier function of IPEC-J2 cells via activating the caspase-3 signaling pathway and inducing cell apoptosis [39], while paclitaxel in IEC-6 cells caused barrier injury via inducing cell apoptosis [40]. Cyclophosphamide was also confirmed to cause barrier dysfunction via the ROS-activated mitochondrial apoptotic pathway [41]. It was also reported that FB1 could induce barrier destruction in swine intestinal epithelial IPEC-1 cells [42]. FB1 was also observed in this study to cause barrier destruction in IEC-6 cells, reflected by decreased TEER, increased paracellular permeability, and reduced production of TJ proteins. However, whether oxidative stress and cell apoptosis are involved in the FB1-caused barrier dysfunction is still unclear. The results from the present study confirmed that FB1 in IEC-6 cells could cause oxidative stress and then lead to barrier destruction via activating both the MAPK and mitochondrial apoptosis signaling pathways. Meanwhile, the three studied polysaccharide samples (i.e., LP, SeLP1, and SeLP2) in the cells could suppress the related signaling pathways. The pretreated cells thus improved the barrier integrity.

Overall, bioactive polysaccharides from various food resources are potential ingredients to maintain intestinal health, as they have a beneficial function in promoting body health and preventing intestinal barrier injury. Accumulating evidence has suggested that polysaccharides can protect intestinal barrier integrity directly by increasing TJ protein expression, regulating inflammation, inhibiting apoptosis, and eliminating oxidative stress, or indirectly by regulating the intestinal microbiota and immunity [9,11,40,43,44]. *Spirulina* polysaccharides thus could mitigate oxidative damage and promote intestinal barrier function [44], while *Ganoderma lucidum* polysaccharides were capable of ameliorating paclitaxel-induced intestinal barrier injury via suppressing cell apoptosis and upregulating TJ proteins [40]. In addition, astragalus and ginseng polysaccharides could enhance piglet growth performance, upregulate TJ proteins, and thus improve the barrier function of the intestine [45], while longan polysaccharides were able to enhance the barrier function of Caco-2 cells via ameliorating claudin-2 expression but increasing ZO-1 expression [46]. It is reasonable to state that both SeLP1 and SeLP2 could protect IEC-6 cells from FB1-induced injury by acting against FB1-induced toxicity and barrier damage.

Polysaccharides’ bioactivity may be altered if they are chemically modified (e.g., acetylation, carboxymethylation, sulfation, and selenylation). It was reported that pumpkin polysaccharides, after an acetylation modification, had a more potent antioxidative effect [47], while *Atractylodes lancea* polysaccharides showed greater immuno-modulation after a carboxymethylation modification [48]. It was also found that the sulfated pacific abalone or *Cyclocarya paliurus* polysaccharides were more active in protecting intestinal barrier integrity [49,50]. Selenylated polysaccharides have been investigated for their effect on the intestine. The Se-containing tea and *Phragmites rhizoma* polysaccharides could alleviate dextran sodium sulfate (DSS)-induced colitis symptoms in model mice and thereby maintain the integrity of the intestinal barrier [19,21]. The results from Zhang and coauthors also indicated that Se-containing *Agaricus blazei Murrill* polysaccharides were more active in alleviating DSS-induced colitis by reducing oxidative stress, and thus showed an ability to improve the intestinal barrier [51]. These published results consistently confirm that the modifications used critically contributed to the polysaccharides’ activity. It was also found that chemical selenylation led to covalent Se conjugation into polysaccharide molecules, thus endowing the selenylated polysaccharides with greater bioactivity in the cells [23,52]. LP, SeLP1, and SeLP2 all belong to polysaccharide substances. Nonetheless, the latter two received a covalent Se conjugation via the conducted chemical selenylation, and thus showed stronger activity in IEC-6 cells than LP.

## 5. Conclusions

The results indicated that the two prepared selenylated LP products had stronger activity than LP in the FB1-exposed IEC-6 cells to alleviate FB1 toxicity via reducing growth inhibition, oxidative stress, and cell apoptosis. The selenylated LP products in the cells were also more effective in combating the FB1-induced barrier disruption, bringing about less LDH release and MMP loss, higher TEER values, lower paracellular permeability, and the upregulated expression of three TJ genes and proteins. Meanwhile, the selenylated products in the cells could suppress the FB1-induced activation of the MAPK and mitochondrial apoptosis signaling pathways. Collectively, the present findings highlight that the covalent selenylation of LP led to Se conjugation into the polysaccharide molecules and then caused the activity increase to mitigate the FB1-induced cell toxicity and barrier loss in IEC-6 cells, especially when a higher selenylation extent was obtained. Thus, the selenylated LP products are potential ingredients for functional foods to maintain intestinal health. It is encouraged to investigate whether Se-rich food ingredients have the potential to protect the intestine from toxin-caused toxicity and other adverse effects.

## Figures and Tables

**Table 1:** Primer sequences used in real-time PCR.

Gene	Primer (5′-3′)
ZO-1	Forward (F)-AGCTGCCTCGAACCTCTACTCTAC
	Forward (R)-GCCTGGTGGTGGAACTTGCTC
ZO-2	F-GATAGCAGCCATCGTGGTCAAGAG
	R-TGCCGACTCCTCTCACTGTAGC
Occludin	F-TGGCTATGGAGGCGGCTATGG
	R-AAGGAAGCGATGAAGCAGAAGGC
Claudin-1	F-GGTGCCTGGAAGATGATGAGGTG
	R-GCCACTAATGTCGCCAGACCTG
Claudin-3	F-GTCGGCCAACACCATCATCAGG
	R-GGCAGGAGCAACACAGCAAGG
GAPDH	F-GGTTGTCTCCTGCGACTTCA
	R-TGGTCCAGGGTTTCTTACTCC

**Table 2:** Expression changes of ZO-1, occludin, and claudin-1 in IEC-6 cells.

Protein	Relative Expression Fold				
	Control	FB1	LP and FB1	SeLP1 and FB1	SeLP2 and FB1
ZO-1	1.0 ± 0.1	0.6 ± 0.1	0.8 ± 0.1	0.8 ± 0.1	0.9 ± 0.1
Occludin	1.0 ± 0.2	0.5 ± 0.1	0.7 ± 0.1	0.8 ± 0.1	0.9 ± 0.1
Claudin-1	1.0 ± 0.2	0.5 ± 0.1	0.6 ± 0.1	0.8 ± 0.1	0.9 ± 0.1

Note: IEC-6 cells were exposed to the medium or polysaccharide products (LP, SeLP1, and SeLP2, 10 μg/mL) for 24 h, and then injured with 40 μmol/L FB1 for 48 h.

**Table 3:** The measured expression changes of the targeted proteins in IEC-6 cells.

Protein	Relative Expression Fold				
	Control	FB1	LP and FB1	SeLP1 and FB1	SeLP2 and FB1
p-JNK/JNK	1.0 ± 0.1	2.0 ± 0.2	1.6 ± 0.1	1.5 ± 0.1	1.3 ± 0.1
p-p38/p38	1.0 ± 0.1	2.5 ± 0.1	2.2 ± 0.1	1.8 ± 0.1	1.5 ± 0.1
Bax	1.0 ± 0.2	1.7 ± 0.1	1.5 ± 0.1	1.3 ± 0.1	1.1 ± 0.1
Bcl-2	1.0 ± 0.1	0.6 ± 0.1	0.7 ± 0.1	0.7 ± 0.1	0.9 ± 0.1
C-caspase-3	1.0 ± 0.2	1.9 ± 0.1	1.9 ± 0.1	1.7 ± 0.1	1.5 ± 0.1
C-caspase-9	1.0 ± 0.1	2.2 ± 0.1	1.9 ± 0.1	1.8 ± 0.1	1.5 ± 0.1
Cytochrome c	1.0 ± 0.1	2.3 ± 0.1	1.9 ± 0.1	1.7 ± 0.1	1.5 ± 0.1

Note: IEC-6 cells were exposed to the medium or polysaccharide products (LP, SeLP1, and SeLP2, 10 μg/mL) for 24 h and injured by 40 μmol/L FB1 for 48 h. C-caspase-3 and C-caspase-9 denote cleaved caspase-3 and cleaved caspase-9, respectively.

## Data Availability

All data are contained within the article.
